# Evaluation of Subjective Appetite Assessment under Free-Living vs. Controlled Conditions: A Randomized Crossover Trial Comparing Whole-Grain Rye and Refined Wheat Diets (VASA-Home)

**DOI:** 10.3390/nu15112456

**Published:** 2023-05-25

**Authors:** Sebastian Åberg, Marie Palmnäs-Bédard, Therese Karlsson, Thérése Hjorth, Kia Nøhr Iversen, Rikard Landberg

**Affiliations:** 1 Department of Life Sciences, Division of Food and Nutrition Science, Chalmers University of Technology, 412 96 Gothenburg, Sweden; 2 Department of Internal Medicine and Clinical Nutrition, Sahlgrenska Academy, University of Gothenburg, 40 530 Gothenburg, Sweden

**Keywords:** appetite response, visual analogue scales (VASs), postprandial satiety, obesity, free-living setting, whole-grain rye, refined wheat

## Abstract

Background: Accurate assessment of self-reported appetite under free-living conditions is warranted to conduct large-scale intervention studies measuring appetite at a feasible cost. However, the performance of visual analogue scales (VASs) for this purpose has not been widely examined. Method: This randomized crossover trial was conducted to evaluate VASs in free-living vs. clinic-based settings and to assess appetite response following hypocaloric whole-grain rye and refined wheat diets. Twenty-nine healthy adults with overweight or obesity continuously answered VAS questions about their perceived appetite from morning to evening. Results: No differences in whole-day VAS scores (primary outcome) between clinic-based and free-living settings were observed, whereas measures of total area under the curve (tAUC) showed increased fullness in clinic-based interventions of 7% (*p* < 0.008) for whole-day responses and 13% (*p* < 0.03) following a snack. Appetite responses for a whole day did not differ between diets whereas rye-based dinners induced 12% (*p* < 0.016) higher fullness and reduced hunger by 17% (*p* < 0.02) irrespective of setting. A reduction in hunger of 15% (*p* < 0.05) was also observed following rye-based vs. wheat-based lunches. Conclusion: The results suggest that the VAS is valid for evaluation of appetite responses between diets under free-living conditions. No difference in self-reported appetite over the whole day was found after whole-grain rye vs. refined wheat-based diets, but there were some suggested differences at certain postprandial periods, in individuals with overweight or obesity.

## 1. Introduction

Obesity is one of the greatest health challenges of today and a leading risk factor for premature death [1]. The imbalance between energy intake and energy expenditure drives the development of overweight, obesity and subsequent diseases, including diabetes and cardiovascular disease [2,3]. Excessive food supply including low nutrient and high energy density foods, such as sugar-sweetened beverages, refined grains and fast foods, is considered to be a major driver in obesogenic development [4,5]. The appetite regulating properties of different foods have been identified as one of the key factors influencing energy intake, and dietary strategies promoting foods that induce feelings of fullness and reduced hunger have the potential to prevent excessive energy intake [6,7]. Traditionally, subjective appetite is measured by ratings of hunger and fullness using 100 mm visual analogue scales (VASs) provided throughout the postprandial period of laboratory test meals [8,9].

A large number of studies have utilized the laboratory test meal procedure and shown a high degree of reproducibility with VASs to measure postprandial appetite [9,10]. However, laboratory test meals and continuous monitoring of study participants are highly resource demanding and require the participants to travel to and reside at the research facilities throughout the study interventions [11]. These methodological challenges can explain why large-scale dietary trials rarely measure subjective appetite. Moreover, studies have shown large inter-individual variability in subjective appetite responses and factors such as perceptions of appetite and habitual eating patterns have been suggested as determinants of inter-individual variation [12,13], calling for crossover study designs. Another challenge when measuring appetite in a controlled clinical setting is the external validity, i.e., generalizability of results, as people in real life are influenced by many psychological cues that do not exist in a clinical setting [9,10]. More feasible and less resource-demanding methods would enable larger intervention groups and allow for studies to examine appetite response to certain foods and their relation to metabolic conditions.

Cereals are important staples and provide >50% of daily caloric intake and represent the most important food source of dietary fiber and plant-based proteins, globally [14,15]. Data from systematic reviews and meta-analyses confirm that consumption of whole-grain foods, compared with refined grain foods, significantly impacts subjective appetite [16]. This might partly explain the inverse associations between WG food intake and risk of overweight, obesity and weight gain over time [17]. Despite the fact that whole grains from different cereals have large differences in content and composition of dietary fiber, other nutrients and bioactive compounds, their effects have rarely been studied separately [18]. 

Wheat and rye are the most common cereals consumed in Northern and Eastern Europe [19]. Rye has the highest content of dietary fiber among all cereals and a contains a variety of bioactive compounds [20]. Several studies with different rye products (porridge, soft bread and crisp breads with different processing conditions and particle sizes) have consistently shown increased satiety compared to isocaloric refined wheat products [16,18,21,22,23,24,25]. However, appetite responses throughout the whole day have not been well studied, nor have appetites in individuals with overweight/obesity or in free-living participants following a rye-based diet. Recently, we showed greater weight and body fat loss following a diet rich in whole-grain rye compared with refined wheat in a 12-week hypocaloric intervention [26]. No differences in appetite response were observed when measured through digital VASs in free-living participants, in contrast with our previous clinic-based studies comparing whole-grain rye and refined wheat foods [22,24,25]. Other large-scale studies in free-living populations with overweight and obesity have also utilized non-validated VASs through mobile apps in measurement of appetite response to habitual diets [27]. To our knowledge, studies that have validated self-reported measurement of appetite under non-monitored free-living conditions compared with clinical conditions are scarce. The aim of this study was to evaluate the performance of VASs in measurements of appetite (fullness, hunger and desire to eat) in a traditional monitored clinical setting compared to under free-living conditions among individuals with overweight and obesity. We tested whether there were any differences in the reporting of appetite measures under free-living conditions vs. clinical setting and if there were any differences between whole-grain rye and refined wheat among individuals with overweight and obesity.

## 2. Materials and Methods

### 2.1. Ethical Statement

This randomized crossover trial was prospectively registered at ClinicalTrials.gov (NCT05004584) and approved by the Swedish Ethical Review Authority on 11 May 2021 (dnr 2021-02489). Recruitment was initiated immediately after approval and the study intervention was conducted between 27 August 2021 and 16 November 2021 at a research clinic in Gothenburg. Individuals interested in participation were given written and oral study information and informed about their ability to withdraw from the study at any time. Written informed consent was obtained from all participants prior to enrollment.

### 2.2. Participants

Participants were recruited through advertisements in local newspapers in Gothenburg and surrounding areas, Facebook pages and through a website for clinical trial advertisements (www.accindi.se, accessed on 16 July 2021) and among previous study participants who have shown interest in future studies. Men and women aged 30–70 years with a BMI of 27–35 kg/m^2^ were eligible for participation and invited to the research clinic for screening and study information. At screening, medical history and lifestyle were examined and fasting blood samples drawn. Eligibility criteria were blood pressure ≤160/105 mmHg, fasting serum triglycerides ≤ 2.6 mmol/L, serum TSH ≤ 3.7 mIE/L and serum LDL ≤ 5.3 mmol/L. Exclusion criteria included smoking or usage of nicotine products, chronic gastrointestinal conditions, thyroid disorder, type 1 diabetes or having undergone major surgery of the gastrointestinal tract, medication for type 2 diabetes or medication for body weight management and dieting or self-reported fluctuations in body weight of more than 10% six months prior to screening. Further reasons for exclusion included pregnancy or lactation, heart attack or stroke in the past 12 months, history of drug or alcohol abuse, allergy or dietary restriction interfering with the study diet or vigorous physical activity >10 h per week. 

### 2.3. Study Design

The present study, referred to as the VASA-home study, was conducted as a randomized crossover trial aiming to compare self-reported appetite responses measured in a controlled clinical setting compared to a standardized free-living setting by using VASs and following a hypocaloric study diet. We also wanted to evaluate the differences in appetite response between whole-grain rye vs. refined wheat diets. Additionally, baseline fecal samples were collected, and blood glucose monitored throughout the study. Participants were randomly assigned to the sequence of five intervention days, separated by a one-week washout, using a Latin square design (“blockrand” package in R). The sequence for each participant was kept in a closed numbered envelope to conceal allocation until enrollment. Participants completed intervention days 1–4 according to location and diet, while the 5th intervention was a clinic-based intervention day with continuous blood sampling and randomization to either wheat or rye-based diets (Table 1). The sequence of interventions 1–5 was completed in random order. There were no dietary restrictions or data collection during washout between interventions days. This paper is reporting data from interventions 1–4 and data collected from continuous glucose monitoring, and microbiome data at baseline will be presented along with other explorative analyses at a later stage. Detailed methodology describing collection of continuous glucose data and physical activity can be found in Supplementary Text S1 and methods for venous blood sampling as well as baseline fecal samples in Supplementary Text S2.

### 2.4. Blinding

Due to the nature of the intervention, the food products differed visually between the dietary treatments, and it was therefore not possible to blind the participants. The cereal products were, however, packed in neutral packaging material. Randomization of intervention sequence was performed by a researcher not involved in the conduct of the study.

### 2.5. Intervention Diets

During all five intervention days, participants followed an individual hypocaloric meal plan providing 1300–2300 kcal/day. Meal plans were calculated based on individually estimated energy requirements with a 500 kcal deficit, using equations developed by [28] and assuming a physical activity level (PAL) of 1.4. Irrespective of allocation and energy requirement, participants consumed a fixed amount of whole-grain rye of 642 kcal or refined wheat cereal products of 652 kcal, providing about one third of the total energy intake instructed in the meal plan for the average participant. However, the energy provided from the cereal products ranged from 28–50% among participants depending on estimated energy requirements. Other foods were adjusted to meet estimated daily energy requirements. The full-day meal plan included a breakfast consisting of cereal puffs with milk, a lunch with tomato soup, crisp bread and cheese/jam, an afternoon snack consisting of crisp bread with cheese/jam and a goulash soup with soft bread and jam/cheese for dinner. Participants were allowed to drink a fixed volume of fluids during intervention days, either coffee, tea or water of their choosing, and instructed to consume the same beverages for all intervention days.

The whole-grain rye cereal products contributed 642 kcal and 32 g of dietary fiber throughout an intervention day. They included 4 slices or 54 g of crisp bread (Wasa), 117 g of soft bread (Lantmännen) and 60 g of rye puffs (Lantmännen). The wheat products were based on refined wheat flour and contributed a total of 652 kcal and 10 g of dietary fiber. They included 5 slices or 66 g of crisp bread (Wasa), 72 g of soft bread (Lantmännen) and 60 g of wheat puffs. Detailed nutritional composition of intervention foods is provided in the Supplementary Materials (Supplementary Table S3). Participants were instructed not to consume any food after 10 p.m. the evening prior to a scheduled intervention day. All foods were provided for the study participants.

### 2.6. Appetite Assessment

Subjective appetite was measured throughout intervention days using a 100 mm VAS. The following three questions were given in random order to evaluate appetite at each timepoint: “How hungry are you?”, “How full are you?”, “How big is your desire to eat?” The participants marked their answer on a 100 mm line anchored at each end with: “not hungry at all” and “very hungry”, “not full at all” and “very full”, “very weak” and “very strong”. 

**Figure 1:**
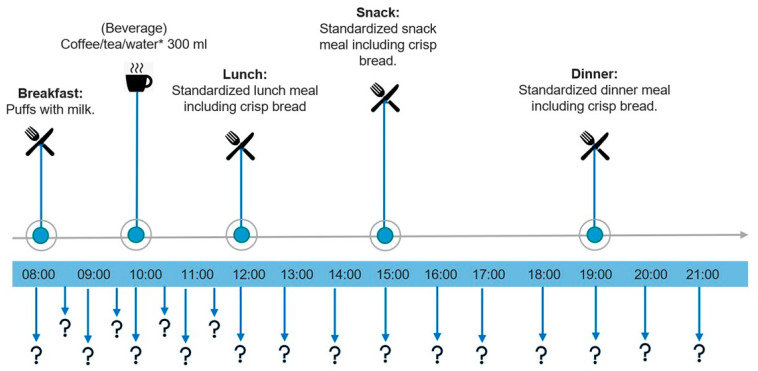
Intervention day overview with appetite questions and meal timings. Question marks (?) indicate timings for VAS questions: fullness, hunger and desire to eat. * Study participants chose 300 mL of coffee, tea or water and consumed the same beverages for all intervention days. VAS: visual analogue scale.

Participants continuously answered questions about their appetite every 30 min from 8:00 to 12:00 and every 60 min from 13:00 to 21:00 (Figure 1). At each of the timepoints when questions needed to be answered, the participants received an automated email through the online software Qualtrics (https://www.qualtrics.com/). Participants followed a hyperlink to Qualtrics.com where the appetite questions were answered. All questions were also provided on paper, giving participants an analogue option and functioning as a back-up if the digital survey failed. A detailed schedule of the timepoints for questions and meals were provided to participants for all intervention days (Figure 1). 

### 2.7. Statistical Analysis

The sample size estimation was calculated with an alpha of 0.05 and power of 0.80 to detect 10% within-group differences in appetite ratings in different settings. The number of participants needed to detect relevant differences between the two study settings has been estimated based on previous interventions studies with similar populations and designs. A total of >16 participants were needed to complete interventions in both settings: free-living and clinic, to detect a contrast with the anticipated variation observed in similar studies before [10]. However, the setting comparison was made after adjustment for dietary treatments of rye and wheat and the variation for the estimated contrasts were likely to be lower compared to a single contrast. Hence, we recruited 29 participants to tolerate a drop-out rate of 30% and allow for secondary comparison of diets and maintain power to address planned exploratory analysis. 

Data were analyzed according to modified intention to treat (mITT), defined as dropouts occurring after randomization but before the intervention start or after the first out of five intervention days were not included in the final analysis. Analyses of appetite in free-living vs. clinic and for rye vs. wheat for the response variables fullness, hunger and desire to eat were performed with linear mixed effects models for repeated measures, using R (version 4.1.2 and the packages lme4, dplyr, tidyverse, emmeans, ggeffects, lmetest). 

In model A, we evaluated the effect of location and diet for whole-day responses (0–780 min) with time, occasion, blood sampling and baseline values as covariates. Subject was included as a random effects variable and the interaction terms diet x occasion; diet x time were evaluated in separate models entitled model A.1 and A.2, respectively. Interaction terms that were not statistically significant were removed in the final model A.3: fullness~diet + location + occasion + time + blood sampling + fullness baseline + (1|subject). The final model was modified to evaluate each of the response variables (fullness, hunger and desire to eat) for both dietary comparison and by location, separately. 

Model B was built to evaluate the effect of location and diet for postprandial periods following breakfast, lunch, snack and dinner and included all above-mentioned covariates, except for the baseline value. Interaction effects were tested and excluded from the final model if not significant. 

In a third model we evaluated the effect of location and diet measured as tAUC. Total area under the curve (tAUC) for appetite responses was calculated using approximating integrals according to the trapezoidal rule [29]. Whole-day response periods and specific postprandial periods were excluded from analysis if missing values accounted for >30%. For tAUC analysis, imputation according to “other day imputation” rules were performed where missing values were <30%. “Other day imputation” was defined as missing data points being imputed with the mean value from corresponding timepoints of remaining intervention days [30].

The third model, model C, included the covariates occasion, blood sampling and subject as random effects variables in the analysis of whole-day response as well as postprandial periods of breakfast–dinner. Physical activity, measured as total step count in the free-living setting and on clinic-based intervention days, was evaluated with subjects modeled as a random variable and occasion as a covariate. Data presented are model-adjusted least-square means with standard error of the mean and considered statistically significant at *p* < 0.05. All values presented were post hoc Bonferroni corrected to take multiple comparisons into account. Descriptive data are presented as mean ± SD. 

## 3. Results

### 3.1. CONSORT Flow Diagram and Participant Characteristics

**Figure 2:**
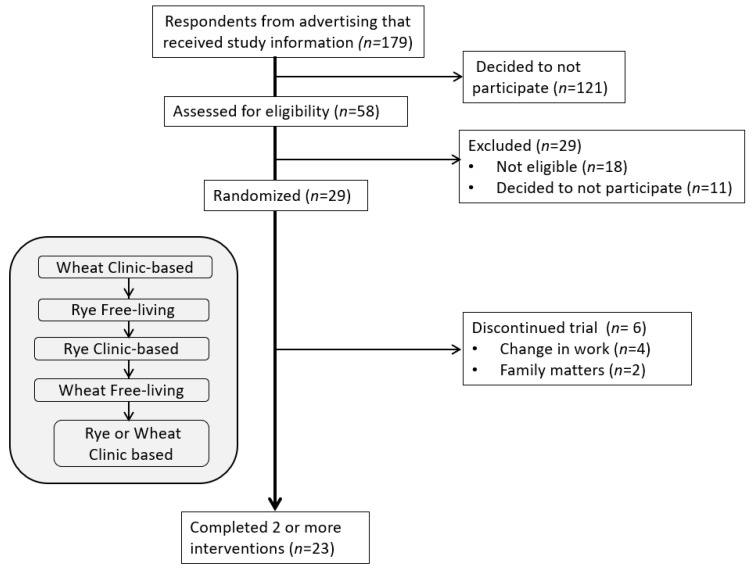
CONSORT flow diagram. Example of sequence order for interventions.

In total, 58 individuals were screened for eligibility to participate in the study and 29 were randomized to intervention order (Figure 2). Six participants discontinued the trial and reasons for not completing the study were change in working hours (*n* = 4) and family matters (*n* = 2). In total, 23 participants (79%) completed two or more out of the five interventions and were included in the analysis of subjective appetite ratings. Participants were predominantly female *(n* = 16) and the mean age of participants was 56 ± 13 years (Table 2). No adverse events were reported.

### 3.2. Appetite Responses

We observed no differences in appetite measures between settings or diets in the fasted state before breakfast. For non-imputed analysis of repeated measures, missing data accounted for 28%, and for tAUC analysis missing data accounted for 18% after imputation.

### 3.3. Contrasts in Appetite between Free-Living and Clinic-Based Interventions

**Figure 3:**
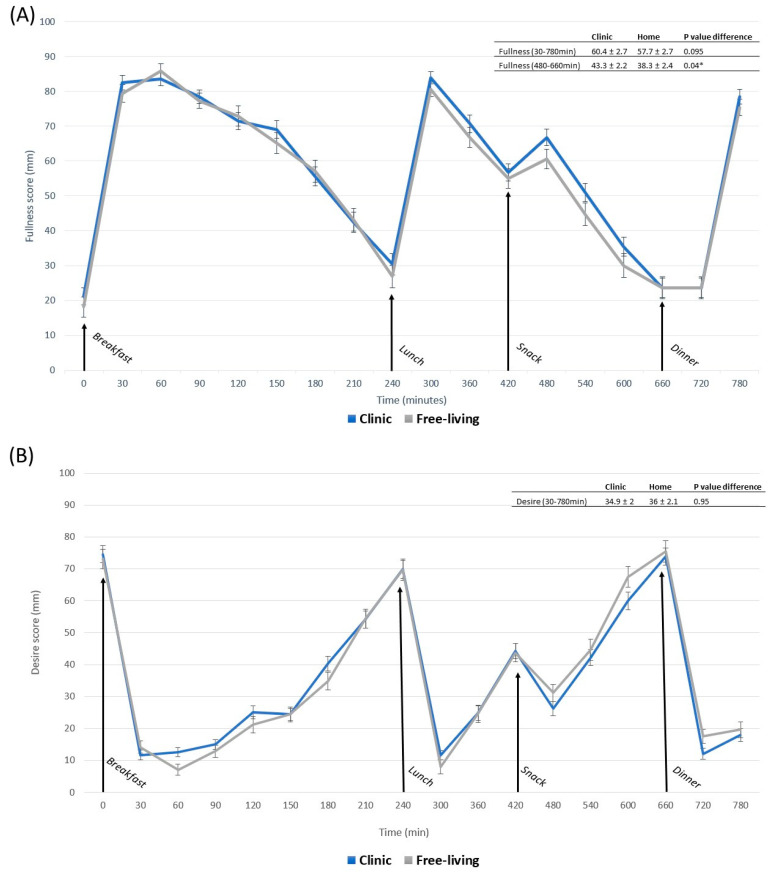

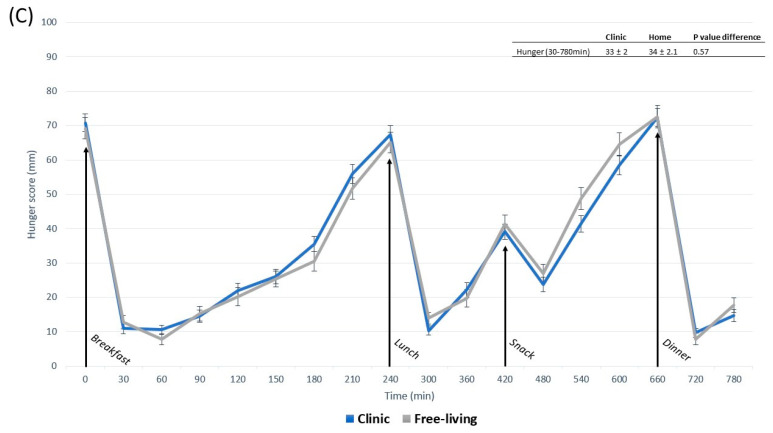
Subjective rating of fullness (**A**), desire to eat (**B**) and hunger (**C**), measured as mm-VAS for free-living compared with clinical setting in postprandial periods: whole day 0–780 min, breakfast 0–240 min, lunch 240–420 min, snack 420–660, dinner 660–780 min. Significant difference (*p* < 0.05) between locations in the same postprandial period is indicated by *. Data are presented as least square means ± SEM, *n* = 23. Whole-day appetite responses were evaluated with the following model: fullness~diet + location + occasion + time + blood sampling + fullness baseline + (1|subject). Postprandial appetite responses were evaluated with the following model: fullness~diet + location + occasion + time + blood sampling + (1|subject). The same models were used to evaluate hunger and desire to eat.

Subjective mean appetite responses (fullness, hunger, desire to eat) did not differ between the free-living setting and clinic when measured as whole-day responses (0–780 min) (Figure 3). Postprandial response following a snack (480–660 min) induced 13% higher fullness for clinic-based interventions compared with free-living interventions (*p* < 0.04). Otherwise, no differences were observed following breakfast (30–240 min), lunch (300–420 min) or dinner (720–780 min). 

**Figure 4:**
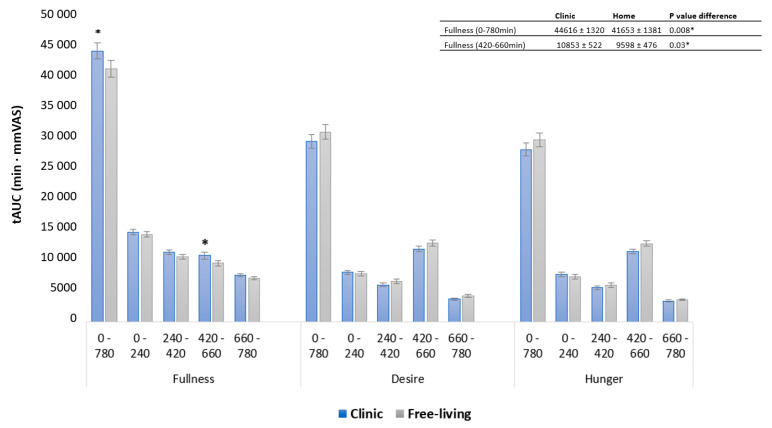
Subjective rating of fullness, hunger and desire to eat, measured as tAUC for free-living compared with clinical setting in postprandial periods: whole day 0–780 min, breakfast 0–240 min, lunch 240–420 min, snack 420–660, dinner 660–780 min. Significant difference (*p* < 0.05) between locations in the same postprandial period is indicated by *. Data are presented as least square means ± SEM, *n* = 23. Whole-day response and specific postprandial appetite responses were evaluated with the following model: fullness~diet + location + occasion + blood sampling + fullness baseline + (1|subject). The same model was used to evaluate hunger and desire to eat.

Hunger and desire to eat, measured as tAUC for the whole-day response, showed no difference between locations. However, fullness values in the clinical setting were 7.1% higher compared with the free-living setting (*p* < 0.008) (Figure 4). The observed difference in fullness following clinic-based snack meals was confirmed by a 13% difference in measures of tAUC (*p* < 0.03) (Figure 4). No other postprandial periods differed in appetite response between free-living and clinic-based settings measured as tAUC. Physical activity, measured as step count, for clinic-based intervention days (4774 ± 455), was lower compared to free-living conditions (6664 ± 477) (*p* < 0.003). In a sensitivity analysis, step count was evaluated as a covariate in all models for clinic and free-living conditions.

### 3.4. Contrasts in Appetite between Whole-Grain Rye- and Wheat-Based Interventions

**Figure 5:**
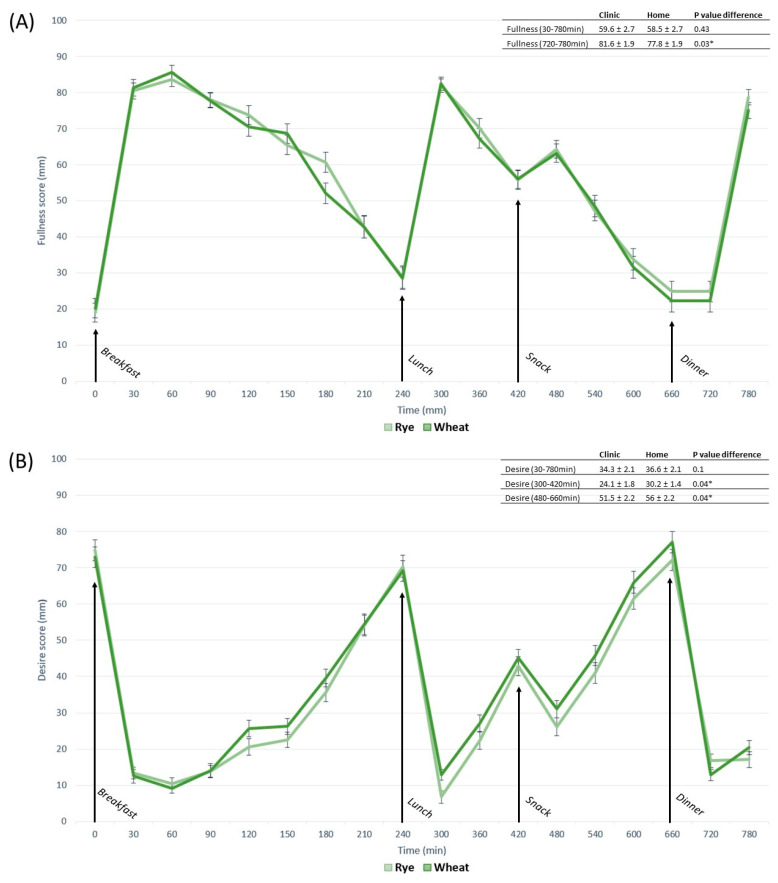

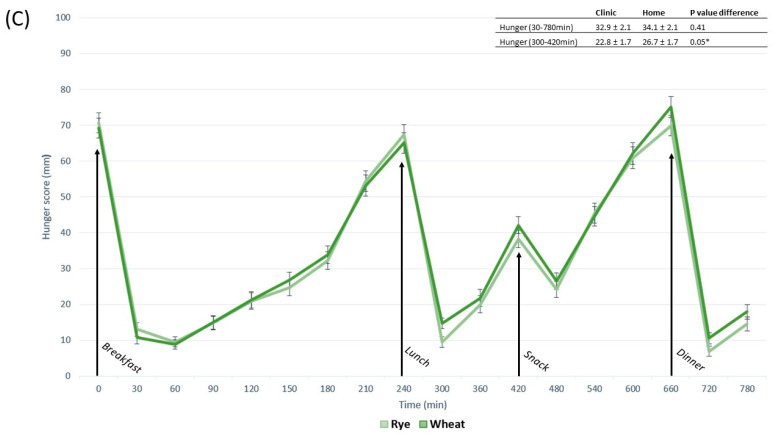
Subjective rating of fullness (**A**), desire to eat (**B**) and hunger (**C**), measured as mm-VAS for rye-based compared with wheat-based meals in postprandial periods: whole day 0–780 min, breakfast 0–240 min, lunch 240–420 min, snack 420–660, dinner 660–780 min. Significant difference (*p* < 0.05) between diets in the same postprandial period is indicated by *. Data are presented as least square means ± SEM, *n* = 23. Whole-day appetite responses were evaluated with the following model: fullness~diet + location + occasion + time + blood sampling + fullness baseline + (1|subject). Postprandial appetite responses were evaluated with the following model: fullness~diet + location + occasion + time + blood sampling + (1|subject). The same models were used to evaluate hunger and desire to eat.

**Figure 6:**
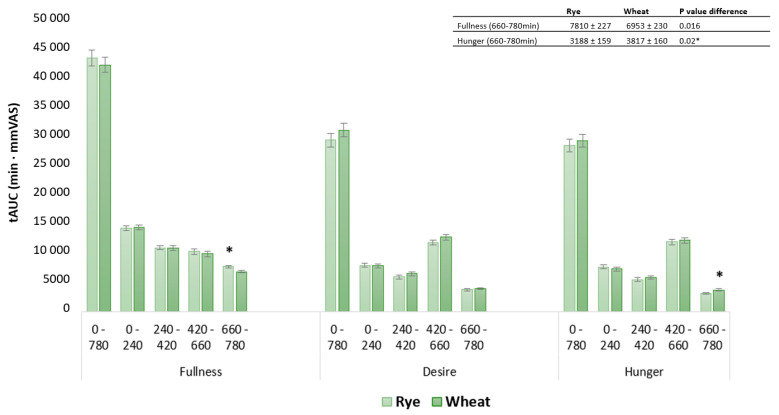
Subjective rating of fullness, hunger and desire to eat, measured as tAUC for rye- and wheat-based diets in postprandial periods: whole day 0–780 min, breakfast 0–240 min, lunch 240–420 min, snack 420–660, dinner 660–780 min. Significant difference (*p* < 0.05) between diets in the same postprandial period is indicated by *. Data are presented as least square means ± SEM, *n* = 23. Whole-day response and specific postprandial appetite responses were evaluated with the following model: fullness~diet + location + occasion + blood sampling + fullness baseline + (1|subject). The same model was used to evaluate hunger and desire to eat.

Appetite response for rye-based interventions did not differ from wheat-based interventions measured as whole-day response. For postprandial periods rye induced higher fullness following dinner 5% (*p* < 0.03) and reduced hunger by 15% (*p* < 0.05) following lunch (Figure 5A,C) compared with refined wheat. Desire to eat was 20% (*p* < 0.04) lower following lunch and 8% (*p* < 0.04) lower following a snack for rye vs. wheat-based meals (Figure 5B). Measures of tAUC showed no differences between diets measured as whole-day responses. However, participants reported 12% higher fullness (*p* < 0.016) and 17% lower hunger (*p* < 0.02) following rye-based dinners compared with corresponding wheat-based meals (Figure 6). No other dietary contrasts were observed in postprandial periods.

## 4. Discussion

In the present study we compared VASs for appetite assessment in participants with overweight or obesity under free-living conditions with traditional appetite assessment conducted in research facilities with full monitoring, which has shown high reproducibility [8]. The need for resource-efficient ways of measuring appetite in a free-living context has increased with large intervention and prospective cohort studies with extensive self-sampling and repeated measures to analyze individual responses in the era of precision nutrition [27]. The results from our comparisons reflect the internal validity of the methods compared since there is no gold standard to compare with, thus external validity cannot be inferred.

We did not find any differences in self-reported whole-day mean appetite response between free-living and clinical settings. The only significant postprandial contrast between free-living and clinical settings was higher fullness in clinic-based interventions following the afternoon snack. This contrast was consistent in mean appetite response as well as tAUC and could be driving the observed difference in whole-day fullness response. 

Our data are essentially in line with data from a recent study where digital VASs in assessment of appetite showed overall concordance between “at home” conditions and traditional laboratory conditions [31]. However, Pasman et al. [31] studied the postprandial period following a 100% whole-grain breakfast in individuals with normal weight.

Although reporting was subjective, no objective measures of compliance can confirm the adherence to diets during free-living intervention days in our study. The lower perceived fullness in the free-living setting in the afternoon may be explained by incomplete consumption of intervention foods. Participants could also be influenced by environmental cues in the free-living setting that do not exist in a clinical setting, including aromas and visual predisposal that induce feelings of hunger and desire to eat [9,10]. We measured physical activity by pedometer in both locations and found a difference corresponding to approximately 75 kcal of energy expenditure [32]. The step count difference was evaluated as a covariate in our statistical models and did not change any results. We speculate that the driving factor behind lower feeling of fullness in free-living circumstances may be related to availability and temptation of other foods in the afternoon. This postprandial period corresponds to 3–7 p.m. and could arguably be more artificial in the clinical setting compared to free-living circumstances. We assume most participants arrived home after work during this period and interacted with family members who were free to eat what they desire, in contrast to study participants who were prohibited from eating until 7 p.m. However, no increase in hunger or desire to eat supports the observed contrast in fullness. Overall, our results show that the VAS in free-living conditions resembles traditional clinic-based methods, with the advantages of reduced costs and study resources and less participant burden. However, the differences in perceived fullness in the afternoon may be addressed in future studies.

Overall, we found no differences for whole-day responses (primary outcome) between diets for either fullness, hunger or desire to eat. However, postprandial responses suggested increased fullness and lowered hunger following specific rye-based meals at certain time windows during the day. Interestingly, appetite response following the last meal of the intervention day showed more consistency with increased fullness and reduced hunger following rye-based meals. A 16% reduction in hunger (*p* > 0.02) measured as tAUC and a 29% borderline significant reduction of mean subjective ratings of hunger (*p* > 0.08) support the observed increase in fullness following rye-based dinners.

Several trials have investigated subjective appetite following whole-grain rye food intake as an acute meal response [21,22,23,25]. These trials demonstrate increased satiety after consumption of whole-grain rye compared with refined wheat as when meals constituted exclusively wheat or rye products. One similar study showed effects in the 4-h postprandial phase when rye contributed two thirds of the total energy content, but no prolonged appetite-suppressing effects after a standardized lunch (not including rye) were demonstrated [24]. Only one of the above-mentioned trials studied populations with overweight and obesity. Hartvigsen et al. [21] demonstrating reduced hunger but no differences in fullness nor prospective consumption following breakfast of 100% rye porridge. In contrast to Isaksson et al. [24], participants continued to report reduced hunger after a standardized lunch, indicating prolonged effects on satiety in this population [21]. We observe similar effects in a similar population (BMI 27–35 kg/m^2^), where consumption of rye products seems to promote satiety later in the day. A possible explanation could be behavioral differences between individuals with obesity and normal weight and perceived satiety could explain observed differences in appetite responses between the above-mentioned trials. In contrast to previous studies, our trial evaluated effects on appetite when commercially available refined grains were substituted with whole-grain rye, as part of a habitual diet and providing only 1/3 of the total caloric intake for the average participant. This is one of the strengths of the trial but also likely the explanation why we cannot demonstrate clear contrasts between diets. 

The relevance of effect sizes seen in our study could be discussed in the context of prospective energy intake. Previous research investigated subjective appetite scores and subsequent energy intake, analyzing 23 randomized controlled trials [6]. A 15 mm change in VAS score for hunger consistently corresponded to a significant change in subsequent energy intake. In the present study, we observed a 4 mm reduction in hunger following rye-based meals compared with wheat, which is not likely to influence prospective energy intake. Future studies should thus investigate subjective fullness and hunger following complex diets including rye cereals and measure subsequent energy intake following rye-based meals. Further, appetite-regulating hormones, glycemia and energy intake are known to vary during the menstrual cycle [33]. Information about menstruation and the use of hormonal contraceptives was not collected and controlled for, which may have influenced subjective appetite reporting of female participants. Additionally, the present study did not have a sufficient number of participants to analyze appetite responses by subgroup, for example, by age, sex or BMI group. Investigations into such relationships could provide complementary insight.

## 5. Conclusions

The findings from the present study in individuals with overweight or obesity suggest that VASs for evaluation of appetite response between diets under free-living conditions can be used interchangeably with controlled clinical conditions due to apparent agreement between the two methods. There were no differences for self-reported appetite responses between rye- and wheat-based diets when compared over the whole day. However, there were indications of reduced hunger and increased feeling of fullness after subsequent whole-grain rye-based vs. refined wheat-based meals, when comparing specific postprandial periods. Future studies need to be conducted to assess the effects on objective measures of appetite response such as specific gut hormones and subsequent meal energy intake.

## Figures and Tables

**Table 1:** Definition of intervention days throughout the study.

(1) Free-living appetite assessment with rye products.
(2) Free-living appetite assessment with wheat products.
(3) A clinic-based appetite assessment with rye products.
(4) A clinic-based appetite assessment with wheat products.
(5) A clinic-based appetite assessment with rye products, with venous blood sampling at 27 timepoints from morning to evening.
(6) A clinic-based appetite assessment with wheat products, with venous blood sampling at 27 timepoints from morning to evening.

**Table 2:** Baseline characteristics of study participants (randomized and completers).

	Randomized	Completers
Characteristics	*n* = 29	*n* = 23
Female (%)	72	70
Age (years)	56 ± 13	55 ± 13
Weight (kg)	87 ± 13	88 ± 13
BMI (kg/m^2^)	32 ± 9	32 ± 10
Systolic BP (mmHg)	130 ± 13	130 ± 12

Data are presented as mean ± SD. Abbreviations: BMI, body mass index; BP, blood pressure.

## Data Availability

The original contributions presented in the study are included in the article. Further inquiries can be directed to the corresponding author and anonymized data will be provided upon reasonable request.

## Supplementary Materials

The following supporting information can be downloaded at: https://www.mdpi.com/article/10.3390/nu15112456/s1, Text S1: Continuous glucose monitors and physical activity; Text S2: Blood sampling and baseline fecal samples; Table S1A,B: Satiety scores measured as 100 mm VAS; Table S2A,B: Satiety scores measured as tAUC; Table S3: Nutritional composition of rye and wheat intervention foods; Table S4: Overview of blood sampling timepoints and meals.
