# Supplementary material for: Evaluation of Subjective Appetite Assessment under Free-Living vs. Controlled Conditions: A Randomized Crossover Trial Comparing Whole-Grain Rye and Refined Wheat Diets (VASA-Home)
Source: Nutrients. 2023 May 25;15(11):2456. doi: 10.3390/nu15112456 (PMC10254760; doi:10.3390/nu15112456)
Supplement: Supplementary file 1 [file nutrients-15-02456-s001.zip › nutrients-2338467-supplementary.pdf]

Supplementary Materials:

Evaluation of Subjective Appetite Assessment under Free-Living vs. Controlled Conditions: A Randomized Crossover Trial Comparing Whole-Grain Rye and Refined Wheat Diets (VASA-Home)

Supplementary Text S1. Continuous glucose monitors and physical activity.

Interstitial blood glucose was measured during all five intervention days through continuous glucose monitors (CGM). The CGM (Abbott Freestyle Libre Pro IQ) sensor was applied at the back of the upper part of the non-dominant arm at the clinic and removed after interventions at the clinic by study personal. Participants were instructed to avoid hot tubes, sauna or other conditions exposing the sensor for high temperature. The sensor is waterproof, hence no restrictions to sanitation or recreational activities were needed. Participants were blinded to glucose data. Physical activity was monitored throughout intervention days using pedometer (Yamax Digiwalker SW-700/701, Yamax, Japan). Participants were instructed to attach the pedometer to the waistband at the hip in the morning 8.00 AM and the step count was registered at the end of each intervention day, at 9.00 PM. Participants were instructed not to look at their step count during intervention days, to avoid behavioral changes.

Supplementary Text S2. Blood sampling and baseline fecal samples.

At the 5th intervention day, participants consumed either wheat or rye-based diets at the clinic and venous blood samples were drawn into a 6 ml EDTA tube and a 4 ml sodium heparin tube by a trained research nurse. Blood samples were collected 10 minutes prior to meals and 15, 35, 65, 95, 125, 155, 185 and 230 minutes after meals, a total of 27 timepoints throughout the intervention day, see supplementary materials (Table 7). An inhibitor cocktail was added to the EDTA tubes to prevent hormone degradation. The inhibitor cocktail was prepared daily by dissolving 1 SIGMAFAST™ Protease Inhibitor tablet (catalogue no. S8820; Sigma-Aldrich Co) in 2.2 ml deionized water containing 5.5 µl 10 mM-dipeptidyl peptidase-IV inhibitor KR-62436 (catalogue no. K4264; Sigma-Aldrich) and dimethyl sulfoxide (Sigma-Aldrich, St. Louis, MO, US). The EDTA and heparin tubes were kept on ice before and after sampling and centrifuged at 4 °C and 2500g for 10min. The blood plasma was transferred to cryotubes and placed in -20 °C for a maximum of 7 days before being transferred to -80 °C for long term storage. Buffy coat and erythrocyte samples were extracted from a fasted EDTA sample (time 0) stored in -20 °C for up to 7 days, before transferred to -80 °C for long term storage.

Baseline fecal samples were collected with EasySampler® fecal collection kit and stored for later analysis. Participants collected the sample within 24 hours of their visit at the clinic and keep it in a cooling bag, with frozen cooling blocks from sampling to delivery at the clinic. Fecal samples that had been stored in household freezer >-18°C were accepted if delivered to the research clinic within 72 hours of sampling. All samples were kept in −20°C freezer for no more than 7 days, and then transferred to −80°C freezer for long term storage.

Supplementary Table S1. Satiety scores measured as 100mm VAS.

	Measure: mm-VAS			
**A)**	**Clinic**	**Free-living**	**P value difference**	**Effect**
Fullness (30–780min)	60.4	57.7	0.095	4,7%
Fullness (30–240min)	63.5	60.7	0.058	4.6%
Fullness (300–420min)	70	65.6	0.026	6.7%
Fullness (480–660min)	43.3 ± 2.2	38.3 ± 2.4	0.04*	13.1%
Fullness (720–780min)	81.5	77.9	0.069	4.6%
Desire (30–780min)	34.9	36	0.525	3.2%
Desire (30–240min)	32.2	32.1	0.96	-0.3%
Desire (300–420min)	27.5	29.4	0.44	6.9%
Desire (480–660min)	51.3	56.2	0.059	9.6%
Desire (720–780min)	14.9	18.3	0.207	22.8%
Hunger (30–780min)	33.4	34.4	0.574	3.0%
Hunger (30–240min)	30.8	30.7	0.94	-0.3%
Hunger (300–420min)	24.4	27.9	0.095	14.3%
Hunger (480–660min)	49.8	54.5	0.07	9.4%
Hunger (720–780min)	11.9	12.6	0.756	5.9%
**B)**	**Rye**	**Wheat**		
Fullness (30–780min)	59.6	58.5	0.43	1,9%
Fullness (30–240min)	62,2	60	0,86	3,7%
Fullness (300–420min)	69,1	66,5	0,13	3,9%
Fullness (480–660min)	41,4	40,2	0,566	3,0%
Fullness (720–780min)	81.6 ± 1.9	77.8 ± 1.9	0.03*	4.9%
Desire (30–780min)	34.3	36.6	0.1	6,7%
Desire (30–240min)	31,9	32,4	0,71	1,6%
Desire (300–420min)	24.1 ± 1.8	30.2 ± 1.4	0.04*	20.2%
Desire (480–660min)	51.5 ± 2.2	56 ± 2.2	0.04*	8.0%
Desire (720–780min)	16,5	16,7	0,942	1,2%
Hunger (30–780min)	32.9	34.1	0.41	3,6%
Hunger (30–240min)	31,2	30,2	0,45	-3,2%
Hunger (300–420min)	22.8 ± 1.7	26.7 ± 1.7	0.05*	14.6%
Hunger (480–660min)	51,1	53,1	0,362	3,9%
Hunger (720–780min)	10,7	13,8	0,0759	29,0%

All values are least-square means.
*For significant contrasts least-square means ± SEM are presented.

Supplementary Table S2. Satiety scores measured as tAUC.

Measure: tAUC				
**A)**	**Clinic**	**Free-living**	**P value difference**	**Effect**
Fullness (30–780min)	44616 ± 1320	41653 ± 1381	0.008*	7.1%
Fullness (30–240min)	14743	14344	0,334	2,8%
Fullness (240–420min)	10853 ± 522	9598 ± 476	0.03*	13.1%
Fullness (420–660min)	10853 ± 522	9598 ± 476	0.03*	13.1%
Fullness (660–780min)	7631	7132	0,197	7,0%
Desire (30–780min)	29721	31234	0,197	-4,8%
Desire (30–240min)	8077	7894	0,679	2,3%
Desire (240–420min)	6032	6621	0,178	-8,9%
Desire (420–660min)	11919	12947	0,109	-7,9%
Desire (660–780min)	3667	4218	0,103	-13,1%
Hunger (30–780min)	28322	29922	0,187	-5,3%
Hunger (30–240min)	7753	7421	0,419	4,5%
Hunger (240–420min)	5570	5980	0,346	-6,9%
Hunger (420–660min)	11541 ± 424	12848 ± 487	0,049*	-10,2%
Hunger (660–780min)	3404	3602	0,491	-5,5%
**B)**	**Rye**	**Wheat**		
Fullness (30–780min)	43708	42561	0,241	2,7%
Fullness (30–240min)	14483	14604	0,74	-0,8%
Fullness (240–420min)	11062	11011	0,893	0,5%
Fullness (420–660min)	10428	10023	0,436	4,0%
Fullness (660–780min)	7810 ± 227	6953 ± 230	0,0159*	12,3%
Desire (30–780min)	29620	31336	0,104	-5,5%
Desire (30–240min)	8030	7941	0,82	1,1%
Desire (240–420min)	6037	6616	0,146	-8,8%
Desire (420–660min)	11969	12898	0,108	-7,2%
Desire (660–780min)	3862	4023	0,594	-4,0%
Hunger (30–780min)	28687	29557	0,422	-2,9%
Hunger (30–240min)	7764	7411	0,334	4,8%
Hunger (240–420min)	5584	5966	0,333	-6,4%
Hunger (420–660min)	12045	12345	0,611	-2,4%
Hunger (660–780min)	3188 ± 159	3817 ± 160	0,0176*	-16,5%

All values are least-square means.
*For significant contrasts least-square means ± SEM are presented.

Supplementary Table S3. Nutritional composition of rye and wheat intervention foods.

(A)	Product weight (g)	Energy (kcal)	CHO (g)	Protein (g)	Fat (g)	Dietary fiber (g)†		
						Total	*Extr.*	*Unextr.*
Extruded rye puffs	100.0	345.4	64.4	8.6	1.5	16.8	6.9	9.9
Rye crispbread	100.0	330.1	59.5	9.8	1.6	17.0	6.7	10.3
Soft rye bread	100.0	218.8	35.8	5.9	3.1	10.6	3.8	6.9
Extruded wheat puffs	100.0	369.2	73.0	12.4	1.3	5.4	2.5	2.9
Wheat crispbread	100.0	382.9	64.5	12.2	6.7	5.6	1.5	4.0
Soft wheat bread	100.0	266.9	48.8	9.9	2.4	3.53	1.03	2.5

(B)	Product weight (g)	Energy (kcal)	CHO (g)	Protein (g)	Fat (g)	Dietary fiber (g)†		
						Total	*Extr.*	*Unextr.*
Extruded rye puffs	60	207.3	38.7	5.2	0.9	10.1	4.1	6.0
Rye crispbread	54	178.3	32.1	5.3	0.9	9.2	3.6	5.6
Soft rye bread	117	255.9	41.9	6.9	3.6	12.4	4.4	8.0
Extruded wheat puffs	60	207.4	43.8	7.4	0.8	3.3	1.5	1.7
Wheat crispbread	66	252.7	42.6	8.1	4.4	3.7	1.0	2.7
Soft wheat bread	72	192.2	35.1	7.1	1.7	2.5	0.7	1.8

(A) Nutritional composition per 100g of product, (B) nutritional composition for products throughout an intervention day.
† Dietary fibre is contributing with 2.0 kcal/g as described by FAO/WHO Expert Consultation on Carbohydrates in Human Nutrition 1997.

Supplementary Table S4. Overview of blood sampling timepoints and meals.

Time	Minutes	Meal
07:50	-10	
**08:00**	**0**	**Breakfast**
08:15	15	
08:35	35	
09:05	65	
09:35	95	
10:05	125	
10:35	155	
11:05	185	
11:50	230	
**12:00**	**240**	**Lunch**
12:15	255	
12:35	275	
13:05	305	
13:35	335	
14:05	365	
14:50	410	
**15:00**	**420**	**Snack**
15:15	435	
15:35	455	
16:05	485	
16:35	515	
17:05	545	

17:35	575	
18:05	605	
18:50	650	
**19:00**	**660**	**Dinner**
19:15	675	
19:35	695	
20:05	725	
20:35	755	

All timepoints for venous blood sampling and meals throughout intervention day 5 with either rye or wheat-based diets.
